# To Inhibit or Enhance? Is There a Benefit to Positive Allosteric Modulation of P2X Receptors?

**DOI:** 10.3389/fphar.2020.00627

**Published:** 2020-05-12

**Authors:** Leanne Stokes, Stefan Bidula, Lučka Bibič, Elizabeth Allum

**Affiliations:** School of Pharmacy, University of East Anglia, Norwich, United Kingdom

**Keywords:** P2X receptor, allosteric modulator, pharmacology, drug discovery, P2X4, P2X7

## Abstract

The family of ligand-gated ion channels known as P2X receptors were discovered several decades ago. Since the cloning of the seven P2X receptors (P2X1-P2X7), a huge research effort has elucidated their roles in regulating a range of physiological and pathophysiological processes. Transgenic animals have been influential in understanding which P2X receptors could be new therapeutic targets for disease. Furthermore, understanding how inherited mutations can increase susceptibility to disorders and diseases has advanced this knowledge base. There has been an emphasis on the discovery and development of pharmacological tools to help dissect the individual roles of P2X receptors and the pharmaceutical industry has been involved in pushing forward clinical development of several lead compounds. During the discovery phase, a number of positive allosteric modulators have been described for P2X receptors and these have been useful in assigning physiological roles to receptors. This review will consider the major physiological roles of P2X1-P2X7 and discuss whether enhancement of P2X receptor activity would offer any therapeutic benefit. We will review what is known about identified compounds acting as positive allosteric modulators and the recent identification of drug binding pockets for such modulators.

## Introduction

Over the last decade we have seen new developments in pharmacological agents targeting P2X4, P2X7, and P2X3 receptors with some candidates entering clinical trials (Keystone et al., 2012; Stock et al., 2012; Eser et al., 2015; Matsumura et al., 2016; Timmers et al., 2018; Muccino and Green, 2019). Drug discovery for other P2X receptors such as P2X1 and P2X2 is somewhat slower with very few selective and potent drugs being identified (Burnstock, 2018). Advances in structural biology have helped move drug design for P2X receptors forward. Accompanying this is the advance in knowledge of the types of physiological responses controlled by this family of ion channels and clinical areas where such drugs may be therapeutically useful. Much emphasis has been placed on the development of antagonist agents and relatively little attention has been on the discovery or development of positive modulators. In this review, we take stock of all the evidence regarding the known physiological roles of the major P2X receptors and present what we currently know about pharmacological agents that can enhance ATP-mediated responses.

**Figure 1:**
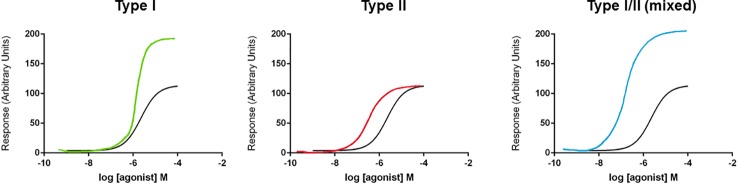
Schematic diagram of types of positive allosteric modulator (PAM) effects on concentration-response curves. Three different types of PAM effect are displayed on concentration-response curves (for illustration purposes only, curves are hand-drawn). A Type I effect is defined as increasing the maximum response without a change in EC_50_ value. A Type II effect is defined as a left-ward shift in the concentration-response curve (and a reduction in the EC50 value) without affecting efficacy (maximum response). A mixed Type I/II effect is defined as both a left-ward shift in concentration-response curve and an increase in efficacy (maximum response).

In receptor function and pharmacology, the word “allosteric” is commonplace. The historical use of this word is discussed by Colquhoun and Lape (2012) and the use of the term allosteric in current pharmacological terminology is used in the context of allosteric modulators, allosteric interactions, and allosteric transitions (Neubig et al., 2003). Allosteric transition describes the mechanism underlying receptor activation; following ligand binding to an orthosteric site, there is conformational communication through the protein to activate the biological response, for example, ion channel pore opening (Changeux and Christopoulos, 2016). Pharmacologically, multiple sites exist on receptor proteins where ligands can bind. While agonists bind at orthosteric sites, modulators act at distinct allosteric (other) sites and typically affect agonist action (Neubig et al., 2003). Allosteric drug interactions can have different outcomes, either positive, negative, or neutral/silent modulatory effects (Neubig et al., 2003; Changeux and Christopoulos, 2016). For positive allosteric modulators (PAMs), these can alter sensitivity to the agonist by shifting the concentration-response curve, alter agonist efficacy by increasing the maximum response, or alter gating kinetics of the ion channel by affecting activation or deactivation (Chang et al., 2010). At the molecular level, it is thought that PAMs reduce the energy barrier for gating thus making it easier for an ion channel to transition into an active, open state (Chang et al., 2010). These two different effects on the agonist actions have been classified by some as Type I (increasing the maximum response or efficacy) and Type II (shifting the concentration-response curve and thus altering agonist EC_50_ values) (Hackos and Hanson, 2017). These two effects may not always be separated with some PAMs exhibiting both effects (mixed Type I/II) (**Figure 1**). The therapeutic beauty of PAMs is their reliance on the presence of the endogenous agonist for activity. Therefore, in disease states where agonist-induced receptor signaling is perhaps defective, PAMs could help to bring those responses back into the “normal” range.

Other ligand-gated ion channels such as the Cys-loop family (GABA_A_ receptor, glycine receptor, nicotinic acetylcholine receptor, and 5-HT_3_ receptor) have many drug binding pockets which are well characterized for both PAMs and negative allosteric modulators (NAMs). The barbiturates and benzodiazepines act as PAMs at the GABA_A_ receptor and are clinically used as sedatives, anti-convulsants, and anaesthetic agents (Solomon et al., 2019). Newer developments have seen multiple PAMs for α_7_ nAchR advance into clinical trials for Alzheimer’s disease, schizophrenia, and ADHD (Yang T. et al., 2017). PAMs for NMDA ionotropic glutamate receptors could be useful for disorders where their hypofunction is implicated (e.g., schizophrenia) (Yao and Zhou, 2017) and PAMs for AMPA ionotropic glutamate receptors could be useful in several cognitive disorders (Lee et al., 2016). For the P2X receptors, only ivermectin, a PAM with activity on P2X4, has been assessed in a pilot Phase 1 clinical trial for alcohol-use disorders (Roche et al., 2016).

We have recently discovered and characterized novel positive allosteric modulators that act on P2X7 and P2X4 receptors (Helliwell et al., 2015; Dhuna et al., 2019) and this has prompted us to ask questions about whether PAMs hold any therapeutic benefit for P2X receptors. We present here a review of the latest literature on P2X receptors focusing on the major identified subtypes P2X1, P2X2, P2X2/3, P2X4, and P2X7 with well-known physiological roles (P2X5 and P2X6 have no assigned physiological roles as homomeric receptors).

## P2X1 Receptor

The P2X1 receptor is a fast desensitizing ion channel activated by ATP, αβ-Me-ATP, βγ-Me-ATP, 2-MeSATP, BzATP, and Ap4A (North and Surprenant, 2000). Human P2X1 was cloned from urinary bladder RNA, and later from human platelets (Longhurst et al., 1996; Sun et al., 1998). Mackenzie et al. first recorded P2X1 currents from human platelets (Mackenzie et al., 1996) and P2X1-dependent Ca^2+^signaling in platelet activation was characterized (Sage et al., 1997). In 1999, P2X1 was stated to have no significant role in platelet aggregation (Takano et al., 1999) however, a later study by Oury et al. demonstrated that P2X1 did act as a positive regulator of platelet responses (Oury et al., 2001). Further to this, a transgenic mouse over-expressing P2X1 in megakaryocytes demonstrated increased αβ-Me-ATP-sensitive Ca^2+^ responses *ex vivo* and more profound platelet shape changes to this agonist (Oury et al., 2003). Tests on these P2X1 over-expressing platelets revealed an increase in collagen-induced aggregation and in the transgenic mouse, an increase in fatal pulmonary thromboembolism was observed compared to wild-type mice (Oury et al., 2003). This work demonstrates that the expression level of P2X1 can modulate platelet aggregation responses. Other studies have investigated the synergy between P2X1 and P2Y1 GPCRs on platelets and it appears that P2X1 activation alone does not induce platelet aggregation (Jones et al., 2014) but that a synergistic activation of P2X1/P2Y1 enables full platelet aggregation. Ca^2+^ influx through P2X1 was deemed critical for this effect (Jones et al., 2014). It is therefore postulated that P2X1 acts as a coincidence detector for released nucleotides and can modulate responses through other platelet receptors (Grenegård et al., 2008; Jones et al., 2014) such as adrenaline and thrombin receptors (Jones et al., 2014) and FcγRIIα (Ilkan et al., 2018). This may be a crucial physiological role for P2X1 to amplify intracellular Ca^2+^-dependent signaling *via* release of nucleotides in an autocrine loop (Ilkan et al., 2018). It is also suggested that P2X1 expressed on neutrophils can be involved in thrombosis (Darbousset et al., 2014) as P2X1^-/-^ mice demonstrated increased polymorphonuclear (PMN) cell accumulation in a laser-injury model which reduced thrombus formation. Thrombosis was restored upon infusion of both platelets and PMNs from wild-type mice whereas infusion of platelets alone did not restore thrombus formation (Darbousset et al., 2014). This was confirmed by using NF449, a selective P2X1 antagonist, demonstrating abolishment of PMN recruitment to the site of injury. With the wealth of evidence showing that P2X1 contributes to platelet aggregation responses, any chronically applied pharmacological agent enhancing P2X1 Ca^2+^ influx in platelets could therefore cause an increased risk of thrombosis, particularly if a positive modulator affects the rate of channel desensitization. Alternatively, acute positive pharmacological modulation may enhance aggregation and clot formation and this may be useful in cases where patients were actively bleeding.

P2X1 is also known to play a role in smooth muscle contraction. ATP is released alongside noradrenaline from sympathetic nerves as a non-adrenergic non-cholinergic (NANC) neurotransmitter. This ATP acts on P2X1 receptors localised on postsynaptic smooth muscle cells (e.g., vas deferens) to contribute to the excitatory junction potential and contractile response (Kennedy, 2015). This work was originally pioneered by Geoffrey Burnstock leading to the accepted notion of purinergic neurotransmission (Burnstock, 2006). ATP is also released from parasympathetic nerves together with acetylcholine and acts on postsynaptic P2X1 in the urinary bladder to induce contractile responses (Kennedy, 2015). It is now thought that P2X1 is the predominant receptor in arterial, bladder, gut, and reproductive smooth muscle (Vial and Evans, 2001). In vascular smooth muscle P2X1 has a role in sympathetic nerve mediated vasoconstriction (Vial and Evans, 2002) and in the renal vasculature, P2X1 is implicated in the regulation of cortical and medullary blood flow by inducing vasoconstriction. In isolated kidneys this autoregulation increases vascular resistance and preglomerular microvascular regulation is thought to stabilize the glomerular filtration rate (Guan et al., 2007). P2X1^-/-^ mice have an impairment in this protective autoregulatory behavior (Inscho et al., 2003). In hypertensive disorders, this renal autoregulation can be defective and purinergic receptors may contribute to the pathophysiology. For example, in Angiotensin-II mediated models of hypertension, there is an increase in vascular resistance causing a reduction in glomerular filtration rate. There is conflicting evidence regarding the expression of P2X1 following chronic administration of Angiotensin-II with some studies reporting an increase (Franco et al., 2011) and other studies reporting a reduction in P2X1 expression (Gordienko et al., 2015).

In the bladder, the purinergic component is likely to be P2X1 and a heteromeric form of P2X1 together with P2X4 (P2X1/4 heteromer) (Kennedy, 2015) and neurogenic contractions can be cholinergic and non-cholinergic. In bladder dysfunction, such as interstitial cystitis, there is an increase in the non-cholinergic mechanisms. In reproductive smooth muscle such as vas deferens, P2X1 induces contractile responses and P2X1^-/-^ mice display defective contraction and are reported to have a deficit in male fertility (Mulryan et al., 2000). Male mice were shown to copulate normally and the reduction in fertility was due to a reduced number of sperm in the ejaculate rather than from sperm dysfunction (Mulryan et al., 2000). Contraction of the vas deferens to sympathetic nerve stimulation in P2X1^-/-^ mice was reduced by 60% (Mulryan et al., 2000). Further studies have shown that the ectonucleotidase NTPDase 1 plays an important role in regulating the activity of P2X1 in the vas deferens by preventing chronic desensitization (Kauffenstein et al., 2014). It has been suggested that pharmacological modulation of P2X1 could be useful in the treatment of male fertility. Blockade of P2X1 may represent a novel target for male contraception but conversely, potentiating P2X1 activity may enhance male fertility associated with defective contraction. Such treatments would need to be tissue-specific to prevent side effects on other physiological responses described.

In terms of therapeutic interventions, enhancement of P2X1 responses could be beneficial in acute platelet aggregation and clot formation, or in boosting vas deferens contractile responses to enhance male fertility (although this would need to be tissue-restricted).

## Positive Allosteric Modulators of P2X1

There are very few studies describing compounds that can potentiate P2X1 responses. Two compounds described as PAMs include MRS2219 and gintonin. MRS2219 is an analogue of PPADS shown to selectively enhance rat P2X1 expressed in *Xenopus oocytes* using two-electrode voltage clamp recordings (Jacobson et al., 1998). MRS2219 had an EC_50_ of 5.9 µM and had no effect on rat P2X2, rat P2X3, or rat P2X4 currents (Jacobson et al., 1998). Gintonin is a water-insoluble non-saponin component of ginseng, made of carbohydrate (mostly glucose), lipids (linoleic acid, palmitic acid, oleic acid, lysophospholipids, phosphatidic acid), and amino acids (Choi et al., 2015) and mainly acts on lysophosphatidic acid (LPA) receptors (Im and Nah, 2013). Using a *Xenopus oocyte* expression system, gintonin was shown to potentiate human P2X1 responses (Sun-Hye et al., 2013) with a similar proposed mechanism to phosphoinositides such as PIP_2_ (Bernier et al., 2008b). Since the exact component of gintonin responsible for P2X1 potentiation is unclear, this needs more investigation in order to be useful in drug discovery. Overall, more information is required on chemicals that can act as selective PAMs at P2X1.

## P2X2 Receptor

The P2X2 receptor is a non-desensitizing ion channel activated by agonists such as ATP, BzATP, and 2-MeSATP (North and Surprenant, 2000). P2X2 was first cloned from rat PC12 pheochromocytoma cells (Brake et al., 1994) and human P2X2 was subsequently cloned from pituitary tissue RNA (Lynch et al., 1999). There is evidence that P2X2 can exist as a homomeric receptor and as a heteromeric receptor in combination with P2X3 (Lewis et al., 1995).

P2X2 is expressed in the inner ear and is thought to play a role in the regulation of hearing. P2X2 mRNA expression increases from embryonic day 12 to postpartum day 8–12 when expression peaks, then decreases to adult levels (Housley et al., 1998). ATP levels in the endolymph are low, P2X2 is activated following exposure to loud noises and contributes to otoprotection whereby ATP is released and acts to reduce the endocochlear potential (Thorne et al., 2004). Part of the adaptive response to loud noise involves an upregulation of P2X2 expression in the rat cochlea (Wang et al., 2003). This upregulation in response to stressors was less in older mice suggesting that this may increase the susceptibility of older animals to noise-induced hearing loss (Telang et al., 2010). In 2013, a human study identified a mutation in *P2RX2* in a Chinese family with inherited progressive hearing loss (Yan et al., 2013). Characterization of this mutation in a HEK-293 cell heterologous expression system revealed that the Val 60 > Leu mutation in P2X2 was not able to respond to ATP (Yan et al., 2013). Individuals carrying the mutation developed severe hearing loss by the age of 20 (Yan et al., 2013). This study also used the P2X2^-/-^ mouse to demonstrate that age-related hearing loss was much greater in these animals than those expressing P2X2 (Yan et al., 2013). In 2014, a second mutation in *P2RX2* was identified in an Italian family with hereditary hearing loss (Faletra et al., 2014). This mutation changed Gly 353 > Arg, a residue in the TM2 domain (Faletra et al., 2014). In 2015, a Japanese study identified a third mutation in *P2RX2* (Asn201 > Tyr) associated with severe hearing loss (Moteki et al., 2015). P2X2 is also thought to play a role in modulating vestibular function. P2X2^-/-^ mice had impaired reflexes in response to sinusoidal rotation when compared to wild-type mice (Takimoto et al., 2018) and were more likely to slip when crossing a narrow beam (Takimoto et al., 2018). It may be advantageous to positively modulate P2X2 responses in the inner ear to increase otoprotection in response to any damage caused by loud noises. This may be of benefit in elderly individuals where P2X2 expression levels are reduced. Similarly, positive modulation of P2X2 may help with balance disorders (e.g., vertigo and Meniere’s disease) and this angle may warrant further investigation.

P2X2 is known to be expressed throughout the hypothalamus and pituitary gland and is involved in the release of hormones such as arginine vasopressin but not oxytocin (Custer et al., 2012). The hypothalamus connects to secretory cells of the anterior pituitary which release chemicals such as luteinising hormone, thyroid stimulating hormone, adrenocorticotrophic hormone (ACTH), growth hormone, prolactin, and follicle-stimulating hormone. This system is intricately balanced and depends on feedback regulation. P2X2 has been directly linked to the enhancement of LH release from the pituitary (Zemkova et al., 2006). The hypothalamus also controls feeding and drinking behavior, reproductive behavior, and temperature regulation and P2X2 is expressed on neurons involved in the regulation of food intake (Collden et al., 2010). Other than regulating hormone secretion directly, P2X2 can play a neuromodulatory role by regulating the release of other neurotransmitters such as glutamate and GABA (Vavra et al., 2011) and in paraventricular neurons, P2X2 can modulate sympathetic activity (Ferreira-Neto et al., 2017). From a therapeutic viewpoint, disorders of the hypothalamus and pituitary gland typically include over-activation (e.g., Cushing’s disease, tumors of NET) rather than under-activation and strategies are needed to limit excessive hormonal secretion. The impact of modulating P2X2 inputs in the hypothalamus is currently unknown.

In 2013, Cao et al. suggested that stimulating P2X2 receptors may be a potential therapeutic strategy for depressive disorders (Cao et al., 2013). Following chronic social defeat stress, mice were found to have lower ATP levels in the brain than control unstressed mice (Cao et al., 2013). Administration of ATP into ventricles (*i.c.v* injection) elicited an anti-depressant-like effect in the immobility test (forced swim test) and the non-hydrolysable ATP analogue, ATPγS, had a larger effect. Administration of ATP together with Cu^2+^ which is known to enhance P2X2 responses, reduced immobility and was interpreted as having an anti-depressant effect (Cao et al., 2013). The proposed mechanism involved ATP release from astrocytes acting on P2X2 receptors in the medial prefrontal cortex and a reduction in P2X2 expression using AAV-shRNA abolished the anti-depressant effect of ATP (Cao et al., 2013). This may provide an interesting mechanistic approach to developing novel anti-depressants, potentially by enhancing ATP release or by enhancing P2X2 signaling.

## P2X2 and P2X2/3 Heteromers

ATP was identified as a neurotransmitter involved in rodent taste perception and areas of the tongue areas involved in taste sensation (circumvallate and fungiform papillae) express both P2X2 and P2X3 receptors (Bo et al., 1999). This was confirmed in a study using transgenic mouse models (Finger et al., 2005). Single gene knockout mice for P2X2 and P2X3 displayed reduced gustatory afferent nerve firing responses to some tastants, however, only in the P2X2/P2X3 double knockout mice were taste responses dramatically affected, losing responses to both sweet and bitter tastants (Finger et al., 2005). This suggested that the P2X2/3 heteromer was responsible for the gustatory neuron signaling to the gustatory cortex and that ATP was crucial for taste signaling. Release of ATP from type II cells, which do not synapse with the gustatory afferent neuron, also appears to be important in taste signaling. In P2X2/3 double knockout mice, tastants failed to release ATP (Huang et al., 2011). As P2X3 is only found in afferent neurons, this indicates a role for homomeric P2X2 receptors in the release of ATP from taste cells. Modulation of either homomeric P2X2 or heteromeric P2X2/3 receptors could therefore affect taste sensation and some P2X3 antagonists have been noted for their suppressive effect on taste in clinical trials (Muccino and Green, 2019).

In terms of therapeutic interventions, enhancement of P2X2 responses could be beneficial in otoprotection or in the modulation of mood. Further mechanistic research into the related physiology and pathophysiology is required to decide if such a pharmacological approach would be viable.

## Positive Allosteric Modulators of P2X2

There is surprisingly little information about the pharmacology of P2X2, in particular human P2X2. In 2011, four derivatives of the anthraquinone dye Reactive Blue 2 were described as having positive allosteric modulator effects at rat P2X2 (Baqi et al., 2011). Reactive Blue 2 is a non-selective antagonist with effect at rat P2X2 and the derivative compound 51 (PSB-10129) increased the maximum response induced by ATP (Baqi et al., 2011). This could be classed as a PAM with Type I effect (**Table 1**). This work demonstrated that lipophilic substitution at certain positions could turn a negative modulator into a positive modulator. Two neurosteroids, dehydroepiandrosterone (DHEA) and progesterone, are known to positively modulate P2X2. DHEA can potentiate both homomeric P2X2 and heteromeric P2X2/3 in recombinant expression models (De Roo et al., 2003; De Roo et al., 2010). Conversely, progesterone potentiates ATP-induced currents in rat dorsal root ganglion neurons and P2X2-expressing HEK-293 cells (De Roo et al., 2010) but not in P2X2/3 expressing *Xenopus oocytes*, suggesting that it is selective for homomeric P2X2 (De Roo et al., 2010). Both of these neurosteroids increased the response to submaximal but not saturating concentrations of ATP suggesting that they affect potency of the agonist but not efficacy. Complete concentration-response experiments would be needed to confirm this, but these could be classed as PAMs with Type II effects (**Table 1**). Testosterone is an endogenous steroid with no potentiating activity at rat P2X2 (Sivcev et al., 2019). However, several synthetic 17β-ester derivatives of testosterone including testosterone butyrate and testosterone valerate, act as PAMs at P2X2 (Sivcev et al., 2019). The testosterone derivatives increased the sensitivity of P2X2 to ATP, reducing the EC_50_ (Sivcev et al., 2019), therefore these PAMs likely have Type II effects (**Table 1**). From this evidence, it is possible that steroids/neurosteroids can act as endogenous positive modulators of P2X2. This could be therapeutically useful, for example, some steroids have been used in treatment of sensorineural hearing loss and Meniere’s disease, as reviewed in (Keiji et al., 2011).

**Table 1:** Chemicals identified as having positive allosteric modulator activity at P2X receptors.

Drug name	Target Receptor	Predicted PAM effect (Type I, II, mixed)	Reference
MRS2219	P2X1 (rat)	Unknown	(Jacobson et al., 1998)
Gintonin	P2X1 (rat)	Unknown	(Sun-Hye et al., 2013)
PIP_2_	P2X1 (rat)	Type I	(Bernier et al., 2008a)
PSB-10129	P2X2 (rat)	Type I	(Baqi et al., 2011)
DHEA	P2X2 (rat) P2X2/3 (rat)	Type II	(De Roo et al., 2003)
Progesterone	P2X2 (rat)	Type II	(De Roo et al., 2010)
Testosterone butyrate	P2X2, P2X4	Type II Mixed Type I/II	(Sivcev et al., 2019)
Ivermectin	P2X4 (rat) P2X4 (human) P2X7 (human)	Mixed Type I/II Mixed Type I/II	(Khakh et al., 1999) (Priel and Silberberg, 2004) (Nörenberg et al., 2012)
Abamectin	P2X4 (rat)	Unknown	(Asatryan et al., 2014)
Selamectin	P2X4 (rat)	Unknown	(Asatryan et al., 2014)
Moxidectin	P2X4	Unknown	(Huynh et al., 2017)
Cibacron blue	P2X4 (rat)	Unknown	(Miller et al., 1998)
Alfaxolone	P2X4 (rat)	Unknown	(Codocedo et al., 2009)
Allopregnanolone	P2X4 (rat)	Unknown	(Codocedo et al., 2009)
THDOC	P2X4 (rat)	Unknown	(Codocedo et al., 2009)
Ginsenoside CK	P2X7 (human), P2X7 (mouse) P2X4 (human)	Mixed Type I/II Mixed Type I/II Mixed Type I/II	(Helliwell et al., 2015) (Bidula et al., 2019a) (Dhuna et al., 2019)
Ginsenoside Rd	P2X7, P2X4	Unknown Unknown	(Helliwell et al., 2015) (Dhuna et al., 2019)
Clemastine	P2X7 (human)	Type II	(Norenberg et al., 2011)
Tenidap	P2X7 (mouse)	Mixed Type I/II	(Sanz et al., 1998)
Polymyxin B	P2X7	Mixed Type I/II	(Ferrari et al., 2004)
Garcinolic acid	P2X7 (human)	Unknown	(Fischer et al., 2014)
Agelastine	P2X7 (human)	Unknown	(Fischer et al., 2014)
GW791343	P2X7 (rat)	Mixed Type I/II	(Michel et al., 2008a)

A Type I positive allosteric modulator (PAM) effect increases efficacy whereas a Type II PAM effect increases sensitivity to agonist seen as a left-ward shift in the concentration response curve. A mixed Type I/II PAM effect represents both features.

## P2X3 Receptor

The P2X3 receptor is a rapidly desensitizing ion channel activated by agonists such as ATP, αβ-MeATP and 2-MeSATP (North and Surprenant, 2000). P2X3 receptors are expressed in sensory neurons where they play a role in nociceptive transmission, taste sensation, bladder distension, and chemoreceptor reflexes (Fabbretti, 2019). P2X3 was cloned from dorsal root ganglion sensory neurons (Chen et al., 1995) and is expressed on afferent C-fibre nerve terminals in peripheral tissues as well as being expressed in central terminals of dorsal root ganglia, as reviewed in (Bernier et al., 2018). Consequently, P2X3 contributes to acute pain signaling and potentially to chronic pain pathways as well (Bernier et al., 2018). P2X3 is expressed in carotid body neurons that regulate the chemoreflex sympatho-excitatory response controlling blood pressure (Pijacka et al., 2016). During the pathophysiology associated with hypertension, P2X3 upregulation can contribute to hyperreflexia and high blood pressure (Pijacka et al., 2016). A recent role for P2X3 has been postulated in chronic cough and airway sensitization due to expression on airway vagal afferent neurons (Abdulqawi et al., 2015; Ford et al., 2015). Continuing this theme of regulating sensory activity, P2X3 is expressed on gustatory sensory neurons and is responsible for taste signaling to the gustatory cortex (Vandenbeuch et al., 2015). As already mentioned, taste signaling involves homomeric P2X3 as well as heteromeric P2X2/3 receptors (Finger et al., 2005). Finally, P2X3 is also documented to have a role in the sensory control of bladder volume. Afferent neurons innervating the bladder express P2X3 and P2X3^-/-^ mice display a reduced bladder voiding frequency (Cockayne et al., 2000). Collectively, this information about the major physiological roles of P2X3 does not present a strong case whereby potentiating ATP-responses at this receptor would be therapeutically useful. There are no known PAMs acting on P2X3 receptors. However, to date nothing is known about the presence of loss-of-function mutations in P2X3 and whether this could be linked to hypo-function of bladder reflexes, for example.

## P2X4 Receptor

P2X4 is a moderately desensitizing ion channel which is activated by ATP, ATPγS, and BzATP (North and Surprenant, 2000). First cloned from rat brain (Soto et al., 1996), P2X4 is widely expressed in the central nervous system, cardiovascular, epithelial, and immune systems. One of the first identified physiological roles for P2X4 was in the cardiovascular system where shear stress-induced ATP release was demonstrated to activate P2X4 on endothelial cells to induce a vasodilatation response (Yamamoto et al., 2000; Yamamoto et al., 2006). Endothelial cells deficient in P2X4 display no flow-regulated Ca^2+^ response or nitric oxide production (Yamamoto et al., 2006). Blood pressure measurements were higher in P2X4^-/-^ mice and the adaptive flow-dependent vascular remodeling response to carotid artery ligation was impaired similar to chronic flow-induced changes in the eNOS^-/-^ mouse (Yamamoto et al., 2006). In humans, a role for P2X4 in regulating flow-dependent vascular tone is postulated and a rare loss-of-function polymorphism was associated with increased pulse pressure (Stokes et al., 2011). It is also thought that P2X4 in the heart could be cardioprotective since cardiac-specific over-expression of P2X4 in mice protected against heart failure (Yang et al., 2014; Yang et al., 2015). In vascular endothelial cells of the brain, P2X4 can also be activated by shear stress and can promote release of osteopontin, a neuroprotective molecule in ischaemic situations (Ozaki et al., 2016). P2X4 was required for ischaemic tolerance in a middle cerebral artery occlusion model of ischaemic stroke (Ozaki et al., 2016).

P2X4 is expressed in epithelial tissues such as salivary glands and bronchiolar epithelium. In the bronchioles, P2X4 is thought to maintain the beating of cilia in the mucus layer, helping to clear the airways of pathogens (Ma et al., 2006). A role has also been described in lung surfactant secretion from alveolar type II epithelial cells (Miklavc et al., 2013). In T lymphocytes, P2X4 can affect T cell activation and migration (Woehrle et al., 2010; Ledderose et al., 2018). In monocytes/macrophages, P2X4 has been linked to release of the chemokine CXCL5 (Layhadi et al., 2018) and the killing of *E. coli* bacteria (Csóka et al., 2018). In the latter study, macrophages taken from the P2X4^-/-^ mouse failed to kill bacteria in response to ATP (Csóka et al., 2018). Potentiation of P2X4 with ivermectin enhanced killing of bacteria and in a mouse model of sepsis, ivermectin improved survival (Csóka et al., 2018).

In the central nervous system, P2X4 is widely expressed on neurons and its role here was recently reviewed (Stokes et al., 2017). Development of a transgenic mouse with a red fluorescent tdTomato under the control of the *P2RX4* promoter confirmed the widespread distribution of P2X4 in the central nervous system (Xu et al., 2016a). In neurons, P2X4 regulates synaptic transmission (Rubio and Soto, 2001; Sim et al., 2006; Baxter et al., 2011) including modulation of GABA release (Xu et al., 2016a). In terms of regulating behavior, P2X4^-/-^ mice exhibit an increased intake of ethanol (Khoja et al., 2018) and this has led to much research on the role of P2X4 in alcohol-use disorders. Treatment with ivermectin counteracts the inhibitory effect of ethanol on P2X4 and can influence the intake of alcohol (Yardley et al., 2012; Franklin et al., 2014). P2X4^-/-^ mice also demonstrate a defect in sensorimotor gating due to dysregulation of dopamine neurotransmission (Khoja et al., 2016). In this study, ivermectin was shown to enhance L-DOPA induced motor behavior suggesting that positive modulation of P2X4 may be a useful adjunct strategy for Parkinson’s disease (Khoja et al., 2016).

Finally, one of the well-known roles for P2X4 involves pathological signaling contributing to neuropathic pain. P2X4 contributes to microglial activation and regulates the release of BDNF which can affect local neurotransmission in the dorsal horn of the spinal cord. Studies have shown that P2X4^-/-^ mice are protected against neuropathic pain (Coull et al., 2005; Ulmann et al., 2008). This work has led to an intensive effort to find antagonists of P2X4 that could be used in the treatment of chronic pain states. Any development of PAMs for therapeutic use would need to be tested for adverse effects on pain states.

## Positive Allosteric Modulators of P2X4

One of the pharmacologically defining features of P2X4 is potentiation by ivermectin (IVM) (Khakh et al., 1999), a derivative of avermectin B1, a macrocyclic lactone produced by *Streptomyces avermitilis*. Through its action on glutamate-gated chloride channels in nematode worms (Cully et al., 1994), IVM is mostly known as a broad-spectrum anti-parasitic agent (Fisher and Mrozik, 1992). IVM also potentiates mammalian GABA_A_ receptors (KrůŠek and Zemkova, 1994) and α_7_-nicotinic acetylcholine receptors (Krause et al., 1998). At P2X4, IVM increases the amplitude of the ATP-induced current at P2X4 with an EC_50_ of ~ 0.25 µM (Priel and Silberberg, 2004; Gao et al., 2015), shifts the EC_50_ for ATP and it changes the desensitization of the P2X4 response (Khakh et al., 1999; Priel and Silberberg, 2004). Therefore, IVM has mixed TypeI/II effects (**Table 1**). IVM may also potentiate the heteromeric P2X4/P2X6 receptor but does not affect P2X2, P2X3, (rodent) P2X7 receptors, or P2X2/3 heteromers (Khakh et al., 1999). Although the crystal structures of closed and ATP-bound state of P2X4 have been solved (Kawate et al., 2009; Hattori and Gouaux, 2012), the IVM-bound structure of P2X4 remains unknown. Priel and Silberberg noted that extracellular application was required for IVM modulation of P2X4 suggesting that IVM does not interact with the intracellular domains (Priel and Silberberg, 2004). It is suggested that IVM most likely partitions into membrane where the lactone ring interacts with the TM domains of P2X4 at the protein-lipid interface. There is also a suggestion that IVM could also affect the trafficking and recycling of P2X4 (Stokes, 2013). Scanning alanine mutagenesis of TM1 and TM2 confirmed that residues near the extracellular surface of the plasma membrane are critical for IVM action (Jelinkova et al., 2006; Silberberg et al., 2007; Asatryan et al., 2010; Popova et al., 2013). Critically, these residues lie either in the extracellular domain (Trp50, Thr57, Ser69, Val60, and Val61) or in the TM2 domain (Asn338, Ser341, Gly342, Leu346, Gly347, Ala349, and Ile356). Asatryan et al. showed that certain amino acids at the interface of the ectodomain and TM2 (Trp46, Trp50, Asp331, Met336) are also involved in determining the selectivity of IVM for P2X4 (Asatryan et al., 2010). Furthermore, the residues lining the edge of the lateral portals are also important (Rokic et al., 2010; Samways et al., 2012; Rokic et al., 2014; Gao et al., 2015). Molecular docking studies have provided important insights and confirmed some of the experimental findings (Latapiat et al., 2017; Pasqualetto et al., 2018).

In various models of disease IVM-dependent increased P2X4 activity might affect alcohol intake, sensorimotor gating, and dopamine-induced motor behavior (Bortolato et al., 2013; Khoja et al., 2016; Khoja et al., 2018; Khoja et al., 2019) implicating P2X4 as a novel drug target for the treatment of alcoholism and psychiatric disorders. IVM has also been shown to have an anti-cancer effect; it kills breast cancer cells through potentiating P2X4/P2X7 signaling (Draganov et al., 2015).

Apart from IVM, other members of the avermectin family that affect P2X4 function are abamectin (ABM), selamectin (SEL), and moxidectin (MOX). ABM is structurally similar to IVM, and similarly potentiated the ATP-induced P2X4 currents. However, at concentrations higher than 3 µM, ABM induced P2X4 responses in the absence of ATP (Asatryan et al., 2014). This may indicate that ABM can act as a direct agonist at higher concentrations. Moreover, in the same concentration range as IVM, ABM was able to antagonize the inhibitory effect of ethanol (100 mM) (Asatryan et al., 2014). In contrast to ABM, SEL is structurally diverse to IVM and was less effective at potentiating P2X4. SEL displayed a lack of efficacy in attenuating the inhibitory effects of ethanol (Asatryan et al., 2014). Lastly, MOX does not possess any saccharide moieties which might add to the increased lipophilicity and a faster penetration across the blood-brain barrier. Similar to IVM and ABM, MOX potentiated the P2X4-mediated currents in *Xenopus* oocytes at 0.5–1 µM and decreased the inhibitory effects of 25 mM (but not 50 mM) ethanol on P2X4 (Huynh et al., 2017). Consequently, this supports the use of avermectins as potential drugs to prevent and treat alcohol use disorders.

Cibacron blue, an anthraquinone sulfonic acid derivative, can potentiate rat P2X4 receptors (Miller et al., 1998). Low concentrations (3–30 µM) resulted in a 4-fold increase in ATP responses however, when tested at 100 µM, cibacron blue was inhibitory at rat P2X4 (Miller et al., 1998). This molecule might represent a novel pharmacophore for the structure-based design of novel allosteric ligands. Similar to P2X2, P2X4 can be modulated by neurosteroids. Alfaxolone, allopregnanolone, and 3α, 21-dihydroxy-5α-pregnan-20-one (THDOC) potentiate rat P2X4 responses in *Xenopus oocytes* and at high concentrations both alfaxolone and THDOC could gate the receptor (Codocedo et al., 2009). The mechanism of potentiation was not investigated in detail but the active neurosteroids could increase response to 1 µM ATP suggesting they may increase receptor sensitivity to agonist (Codocedo et al., 2009). A study found that testosterone 17β-ester derivatives such as testosterone butyrate and testosterone valerate could enhance P2X4 responses (Sivcev et al., 2019) by increasing receptor sensitivity to agonist (mixed Type I/II effect).

Recently, our lab identified ginsenosides of the protopanaxdiol series as positive allosteric modulators at P2X7 and P2X4 receptors (Helliwell et al., 2015; Bidula et al., 2019b; Dhuna et al., 2019). By using a plethora of techniques, including fluorescent YOPRO-1 dye uptake assays in stable cell lines over-expressing human P2X4, calcium assays, and electrophysiology, we demonstrated that two ginsenosides, CK and Rd, show ~2-fold potentiation of ATP-responses at P2X4 (Dhuna et al., 2019) which could be classed as a mixed Type I/II effect (**Table 1**). Enhancement of P2X4 is less than enhancement of P2X7 and our docking studies have predicted that while the interacting amino acid residues are similar in both receptors, subtle differences in the binding pocket might modify the way these ginsenosides bind to P2X4 (Dhuna et al., 2019). However, this may also provide novel pharmacophore information for development of selective PAMs.

In terms of therapeutic interventions, enhancement of P2X4 responses could be beneficial in hypertension (to reduce blood pressure *via* vasodilation), sepsis, Parkinson’s disease, or in alcohol-use disorders. Thus, more research is justified to investigate PAMs and to determine their mechanisms of action both *in vitro* and *in vivo*.

## P2X7 Receptor

P2X7 is a non-desensitizing ion channel activated by ATP and BzATP (North and Surprenant, 2000). First cloned in 1996 (Surprenant et al., 1996), P2X7 is expressed in immune cells such as monocytes, macrophages, NK cells, lymphocytes, and neutrophils (Di Virgilio et al., 2017) and has predominantly been characterized by the intracellular signaling pathways that it regulates (Bartlett et al., 2014). P2X7 requires high concentrations of ATP for activation and displays a somewhat unique secondary pore-forming phenomena allowing movement of organic molecules across the cell membrane. The physiological function (and substrates) of this secondary pore pathway is currently unclear, however, it is likely to play a role in many P2X7 signaling events (Di Virgilio et al., 2018). Activation by high concentrations of ATP is consistent with its role in inflammation, where ATP can be released from stressed or damaged cells and functions as a damage-associated molecular pattern (DAMP) (Di Virgilio et al., 2017). Often, activation of P2X7 at these inflammatory sites can be detrimental and this may contribute to the pathophysiology of a plethora of inflammatory disorders. Conversely, it is possible that P2X7 activation may be beneficial in the defence against intracellular pathogens and cancerous cells.

P2X7 is a known regulator of immune cell mediator secretion. Multiple studies have demonstrated secretion of cytokines from the IL-1 family (IL-1β, IL-1α, IL-18) in response to P2X7-dependent activation of the NLRP3-caspase-1 inflammasome (Giuliani et al., 2017). Other cytokines such as those relying on cleavage by metalloproteinases (e.g., TNF-α) are also released following P2X7 activation as well as other cell surface proteins (e.g., L-selectin, VCAM-1, CD23, and CD14) which are shed (Pupovac and Sluyter, 2016). The particular cytokines released by P2X7 may depend on the cell type under examination, for example, in T lymphocytes, P2X7 can contribute to IL-2 production and secretion (Yip et al., 2009). Current knowledge may only be the tip of the iceberg as other immune cell types have not been rigorously examined. P2X7 can also contribute to the regulation of various caspase-dependent and -independent cell death pathways, including autophagy, necrosis, pyroptosis, and apoptosis, governing the homeostatic turnover of cells and modulating immunity to pathogens (Di Virgilio et al., 2017). Although the major physiological roles of P2X7 may be complex to pin down, it is clear that this receptor is involved in inflammation and infection. For a comprehensive overview of the pathophysiological roles of P2X7 in this context, please refer to (Di Virgilio et al., 2017; Burnstock and Knight, 2018; Savio et al., 2018).

P2X7 activation is important in the defence against intracellular bacteria such as *Chlamydiae*, *Porphyromonas gingivalis*, and *mycobacteria* species. P2X7 promotes the acidification of intracellular organelles, phospholipase D activation and decreases bacterial load (Coutinho-Silva et al., 2001; Coutinho-Silva et al., 2003; Darville et al., 2007). Consequently P2X7^-/-^ mice are more susceptible to vaginal infection by *Chlamydiae* (Darville et al., 2007). P2X7 plays an important role in defence to *P. gingivalis*, the causative agent of periodontitis, *via* regulation of inflammasome activation (Choi et al., 2013; Hung et al., 2013; Park et al., 2014). The role of P2X7 in mycobacterial infections appears to be strain specific. On the one hand, loss-of-function in P2X7 may contribute to enhanced susceptibility to pulmonary and extra-pulmonary tuberculosis in humans (Fernando et al., 2007). P2X7 participates in the elimination of the intracellular bacteria *via* phospholipase D activation and host cell apoptosis (Fairbairn et al., 2001; Placido et al., 2006; Fernando et al., 2007; Singla et al., 2012; Areeshi et al., 2015; Wu et al., 2015). Conversely, mice infected with hypervirulent mycobacterial strains cannot effectively control the infection and P2X7 contributes to the severity of inflammation and propagation of bacterial growth (Amaral et al., 2014). With such hypervirulent strains, mice deficient in P2X7 were better protected against the infection (Amaral et al., 2014).

A role for P2X7 in the immune response to parasites *Leishmania amazonensis*, *Toxoplasma gondii*, *Plasmodium falciparum* (Salles et al., 2017), and *Entamoeba histolytica* (Mortimer et al., 2015) is also becoming clear. Macrophages infected by *L. amazonensis* can reduce their parasitic load *via* the P2X7-dependent production of the mediator leukotriene B4 (LTB_4_) (Chaves et al., 2009; Chaves et al., 2014). Again, P2X7^-/-^ mice were more susceptible to infection (Figliuolo et al., 2017). P2X7 activation can drive the elimination of *T. gondii via* the production of ROS, acidification of intracellular organelles (Correa et al., 2010; Moreira-Souza et al., 2017), and secretion of pro-inflammatory cytokines (Miller et al., 2011; Miller et al., 2015; Correa et al., 2017; Huang et al., 2017). Therefore, for parasitic infections, enhancing P2X7 responses may be therapeutically beneficial.

In models of infection, it is less clear how P2X7 affects outcomes in cases of sepsis. In a murine model of sepsis, P2X7^-/-^ mice had a better chance of survival (Santana et al., 2015; Wang et al., 2015). Pharmacological inhibition using the P2X7 antagonists A-740003 or Brilliant Blue G, resulted in increased survival, downregulating inflammation and maintaining mucosal barrier integrity (Greve et al., 2017; Savio et al., 2017; Wu et al., 2017). A risk genotype of human P2X7 containing a known gain-of-function haplotype (P2X7-4.1 in (Stokes et al., 2010)) was increased in a cohort of sepsis patients (Geistlinger et al., 2012). Recent work shows P2X7 activation in human monocytes compromised subsequent NLRP3 inflammasome activation by bacteria and contributed to mitochondrial dysfunction (Martinez-Garcia et al., 2019). Impairment of NLRP3 was associated with increased mortality in sepsis patients (Martinez-Garcia et al., 2019) suggesting that P2X7 activation plays a detrimental role in sepsis. In the same study, the murine CLP model was used to test the role of activation of P2X7 *in vivo* prior to induction of sepsis and the authors documented an increased mortality (Martinez-Garcia et al., 2019) However, opposing studies using the murine model suggest that P2X7 could be protective within sepsis and demonstrated increased mortality in P2X7^-/-^ mice (Csoka et al., 2015). This issue of the role P2X7 plays during sepsis needs further investigation for further progress to be made.

P2X7 has been implicated in the immune response to several viruses including; vesicular stomatitis virus (VSV), influenza virus, dengue virus, and HIV. In the case of VSV and dengue virus, P2X7 plays a beneficial role, with ATP-induced signaling resulting in decreased viral replication (Correa et al., 2016; Zhang et al., 2017). Conversely, evidence points towards a detrimental role for P2X7 in influenza and HIV infections. In the case of influenza, P2X7 deficiency protected against a lethal dose of the virus due to a reduction in inflammatory mediators and reduced neutrophil recruitment (Leyva-Grado et al., 2017). More recently, administration of the P2X7 antagonist AZ11645373 or probenecid [an approved drug known to inhibit P2X7 (Bhaskaracharya et al., 2014)], improved survival and recovery to pathogenic influenza infection in a murine model (Rosli et al., 2019). For HIV infection, pharmacological inhibition of P2X7 could limit replication of the virus within macrophages, and prevent virion release (Hazleton et al., 2012; Graziano et al., 2015). With viral infections, enhancing P2X7 responses may only be beneficial in certain cases and much more work is needed to fully understand potential therapeutic interventions.

The role of P2X7 in anti-fungal immunity is currently under-explored but studies have reported that P2X7 is not involved in scavenging *Candida albicans* and in the production of IL-1β in response to yeast infection (Hise et al., 2009; Perez-Flores et al., 2016). Xu et al*.*, demonstrated that invariant natural killer T (iNKT) cells release ATP and induce Ca^2+^ signaling in dendritic cells, which stimulates the production of prostaglandin E2, recruitment of neutrophils, and reduced *C. albicans* infection (Xu et al., 2016b). A more recent study showed that P2X7 was critical for the induction of adaptive immune responses to *Paracoccidioides brasiliensis* and survival (Feriotti et al., 2017).

P2X7 is also expressed in glial cells within the central nervous system including microglia, oligodendrocytes, astrocytes, and there is some (often debated) evidence for expression in neurons. In the CNS, more P2X7 plays potential physiological roles in neuronal axonal growth and modulation of neurotransmitter release but also participates in neuroinflammation (Bartlett et al., 2014). Under pathological conditions or following damage to the CNS, a significant amount of ATP can be released which contributes to neuroinflammation. It is predominantly activation of P2X7 on microglia that stimulates the production of pro-inflammatory mediators and ROS. This neuroinflammation combined with an increase in cell death stimulates an environment whereby extracellular ATP concentrations are further enhanced, stimulating more cell death, including the death of neurons. Dysregulated P2X7 activation has therefore been touted as a key contributor to the pathophysiology of Alzheimer’s disease, Parkinson’s disease, and multiple sclerosis, among others. Genetic ablation of P2X7 dampens neuroinflammation and enhances the clearance of amyloid-β plaques (Mclarnon et al., 2006; Ryu and Mclarnon, 2008; Ni et al., 2013). Such neuroinflammatory responses may also be involved in psychiatric disorders, as reviewed in (Bhattacharya and Biber, 2016). Indeed, a gain-of-function haplotype of human P2X7 has been repeatedly linked to various psychiatric conditions including bipolar disorder, major depressive disorder, and anxiety disorders (Czamara et al., 2018; Deussing and Arzt, 2018). Current drug development programs are focused on testing CNS penetrant P2X7 antagonists for psychiatric conditions (Bhattacharya and Ceusters, 2020).

In the cardiovascular system, P2X7 participates in inflammation, cell metabolism, and cell death and therefore impacts ischemic heart disease, stroke, and vascular diseases such as atherosclerosis, hypertension, thrombosis, and diabetic retinopathy. P2X7 activation can contribute to cardiac dysfunction in myocardial infarction due to its role in inflammation which can facilitate sympathetic sprouting and arrhythmia (Lindholm et al., 1987; Yang W. et al., 2017). Notably, activation of P2X7 by the synthetic agonist BzATP can upregulate the secretion of nerve growth factor (NGF), which may be linked to enhanced sympathetic hyper-innervation and sprouting (Yin et al., 2017). P2X7 is upregulated at the site of infarct and can promote the activation of the NLRP3 inflammasome and the release of inflammatory IL-1β within the ventricles (Yin et al., 2017). Inhibiting P2X7 was demonstrated to promote cardiac survival, suppress T cell mediated immune responses, and limit the risk of rejection (Vergani et al., 2013). P2X7 generally contributes to excessive inflammation in the vasculature and is implicated in several vascular diseases *via* IL-1β production and production of matrix metalloproteases (MMPs) which contributes to the pathophysiology of atherosclerosis (Lombardi et al., 2017).

In the lung, P2X7 is a potential target for lung hypersensitivity associated with chronic inflammatory responses. Targeting P2X7 may control IL-1β-induced lung fibrosis and silicosis (Moncao-Ribeiro et al., 2014). Inhibiting P2X7 on dendritic cells and eosinophils could be beneficial in the treatment of allergic asthma, and the anti-histamine, oxatomide, has been suggested to be a P2X7 antagonist (Yoshida et al., 2015). Further, P2X7 has been associated with pulmonary oedema and with emphysema, the latter linked to the inhalation of cigarette smoke inducing ATP release (Lucattelli et al., 2011). P2X7 is connected to the recruitment of inflammatory cells to the lung during injury, particularly neutrophils, which further enhance lung injury. In this case, deletion or inhibition of P2X7 appeared to be protective within the lung. Therefore, with both cardiovascular and lung disorders, enhancement of P2X7 responses would not likely be of any advantage and most research is focused on testing P2X7 inhibitors.

In bone, P2X7 is involved in osteogenesis (Sun et al., 2013) and the development of mature osteoblasts (Gartland et al., 2001; Ke et al., 2003; Panupinthu et al., 2008). In these cells P2X7 participates in functions such as production of lipid mediators, induction of transcription factors, propagation of intercellular calcium signaling between osteoblasts and osteoclasts, and intracellular signaling in response to fluid shear stress (Gartland et al., 2001; Jorgensen et al., 2002; Liu et al., 2008; Okumura et al., 2008; Panupinthu et al., 2008; Gavala et al., 2010). This positive role of P2X7 in the maintenance of bone strength is supported by studies utilizing mesenchymal stem cells (MSCs) taken from post-menopausal women, where bone mineralization and osteogenic differentiation were impaired (Noronha-Matos et al., 2014). Notably, administration of BzATP *in vitro* could restore these functions, indicating an important role for P2X7 in driving the formation of bone. P2X7 has been suggested to be involved in differentiation of osteoclasts (Barbosa et al., 2011) and the generation of multinucleated cells, however some evidence from P2X7^-/-^ mice shows that this is a redundant process not solely reliant upon P2X7 (Gartland et al., 2003; Ke et al., 2003; Agrawal et al., 2010). Treatment of osteoclasts with BzATP or high ATP to stimulate P2X7 can increase bone resorption and this effect is lost in P2X7^-/-^ mice (Jiang et al., 2000; Armstrong et al., 2009; Hazama et al., 2009). Miyazaki et al. demonstrated that bone resorption relies on intracellular (ATP) and mitochondrial function (Miyazaki et al., 2012). In this study treatment of bone marrow-derived osteoclasts with extracellular ATP resulted in decreased survival and resorption (Miyazaki et al., 2012). The differences between may be due to species specific differences or genetic variation, but a fine balance between P2X7 activation/inactivation must be maintained to ensure optimal osteoclast function (Donnelly-Roberts et al., 2009; Bartlett et al., 2014).

Skeletal muscle is required for numerous structural and biological functions within the body. When muscles are stimulated, they release small amounts of ATP which propagates intracellular Ca^2+^ signaling and downstream biological effects. However, when muscles are damaged, much larger concentrations of ATP are released, triggering an inflammatory response. An acute inflammatory response is essential for muscle repair and regeneration, but prolonged inflammation can result in muscular dystrophies (Tidball and Villalta, 2010). P2X7 expression is increased in the muscles of Duchenne’s muscular dystrophy patients and in murine models of muscular dystrophy (Young et al., 2012). P2X7 can contribute to sterile inflammation by promoting the release of inflammatory mediators from dystrophic muscles (Rawat et al., 2010) or contribute to deregulated homeostasis in dystrophic muscles (Young et al., 2015). In the MDX model of muscular dystrophy, P2X7 deficiency reduced dystrophic symptoms such as decreased muscle structure and increased inflammation, whilst promoting expansion of T regulatory cells known to suppress dystrophic muscle damage (Sinadinos et al., 2015). Surprisingly, cognitive and bone improvements were also noted in these animals (Sinadinos et al., 2015). As for many of the disorders linked to excessive inflammation, enhancement of P2X7 responses in this context would be predicted to be detrimental.

Finally, the role of P2X7 in cancer development and progression will be considered. A feature of some tumor cells are their high levels of P2X7 expression, which can mediate cell proliferation, or cell death depending upon the type of tumor, the variant of P2X7 expressed and potentially, the cellular environment. Tumors often produce high concentrations of extracellular ATP within the tumor core which would enable P2X7 signaling (Burnstock and Knight, 2018). P2X7 antagonists have been suggested as potential anti-metastatic agents by reducing tumor cell proliferation. Conversely, it is thought that activating P2X7 on tumor cells could result in cell death.

First of all, considering the role of P2X7 in tumor cell proliferation, expression of P2X7 on tumor cells is associated with accelerated tumor growth (Adinolfi et al., 2012). γ-irradiation can induce the release of ATP from B16 melanoma cells, which results in proliferation and tumor growth (Hattori et al., 2012). Inhibition of P2X7 with AZ10606120 reduced proliferation of human pancreatic duct adenocarcinoma and human neuroblastoma cells *in vitro* (Amoroso et al., 2015; Giannuzzo et al., 2016). Furthermore, AZ10606120 reduced neuroblastoma tumor growth in nude mice (Gomez-Villafuertes et al., 2015). P2X7 can contribute to the metastasis of human lung cancer cells, and P2X7 inhibition significantly decreased the migration of cancer cells transplanted into immunodeficient mice (Takai et al., 2014; Schneider et al., 2015). Emodin, a natural product antagonist of P2X7, could reduce the invasiveness of a highly invasive breast cancer cell line and ATP could elicit an increase in cell migration and metastasis in another breast cancer cell line (Jelassi et al., 2013; Xia et al., 2015). P2X7 expression is being used post-operatively as a prognostic indicator for survival in renal cell carcinoma patients (Liu et al., 2015). Expression of a non-pore functional P2X7 (nfP2X7) was found in pathological specimens from prostate cancer patients and was not observed in normal patients suggesting this as a possible biomarker of prostate cancer (Slater et al., 2004). A more recent study suggests nfP2X7 is broadly expressed on many tumor cells (Gilbert et al., 2019). While it is unclear which splice variant encodes nfP2X7, antibodies recognizing this different form of P2X7 have been tested in a Phase I safety and tolerability trials for basal cell carcinoma (Gilbert et al., 2017). P2X7 plays a deleterious role in osteosarcoma and can contribute to cancer-induced bone pain (Giuliani et al., 2014; Falk et al., 2015). With gliomas, P2X7 activation is linked to an increase in inflammation, intracellular calcium signals, and tumor cell migration (Morrone et al., 2016).

P2X7 may play a role in the host immune response to tumor cells. In 2015, Adinolfi et al. reported that tumor progression was accelerated in mice lacking P2X7 (Adinolfi et al., 2015). Expression of P2X7 on host immune cells was critical for controlling the anti-tumor immune response (Adinolfi et al., 2015). In P2X7^-/-^ mice, an immunocompromized tumor infiltrate was characterized with few CD8^+^ T cells and an increased number of T regulatory cells (De Marchi et al., 2019).

Alternatively, P2X7 activation may be important in the eradication of certain types of tumor. P2X7 activation has been demonstrated to induce apoptosis in acute myeloid cells but not haematopoietic stem cells (Salvestrini et al., 2017). A useful review of the literature is presented by Roger et al., where therapeutic strategies for solid tumors including promoting the cytolytic effect of ATP, are discussed (Roger et al., 2015). Many *in vitro* studies have shown that ATP or BzATP can be cytotoxic to tumor cells (Roger et al., 2015) and some have shown an effect of ATP on melanoma *in vivo* (White et al., 2009). Exploitation of the high level of expression of P2X7 on tumor cells to stimulate tumor cell death is an option explored by (De Andrade Mello et al., 2017). This study used hyperthermia to enhance membrane fluidity and potentiate ATP-induced cytotoxicity *via* P2X7 in colon cancer cells *in vitro* (De Andrade Mello et al., 2017) although such an approach has not yet been tested *in vivo*. This does highlight the possibility of using positive allosteric modulators to provide a similar enhancement of P2X7-induced cell death.

Summarising therapeutic interventions, enhancement of P2X7 responses could be beneficial in infectious diseases (particularly with intracellular bacteria and parasites) to boost microbial defences, in anti-tumor immunity, and induction of tumor cell death. More research is required to develop selective PAMs and to determine their mechanisms of action both *in vitro* and *in vivo*.

## Positive Allosteric Modulators of P2X7

A number of chemically distinct molecules have been suggested to act as positive modulators of P2X7. Clemastine, a first-generation anti-histamine, acts to positively modulate P2X7 in mouse and human macrophages (Norenberg et al., 2011). The combination of clemastine and ATP could enhance P2X7-mediated whole-cell currents, Ca^2+^ entry, pore-formation, and IL-1β release from human monocyte-derived macrophages and murine bone marrow-derived macrophages (Norenberg et al., 2011). Clemastine is thought to bind extracellularly to an allosteric site and concentration-response experiments using whole-cell recordings revealed an effect on sensitivity to agonist but not efficacy (Norenberg et al., 2011) therefore showing a Type II PAM effect (**Table 1**). There have been few studies so far investigating the effects of clemastine-induced potentiation of P2X7 in a biological setting. In a murine model of amyotrophic lateral sclerosis (ALS), a short treatment with clemastine (from postnatal day 40 to day 120) could delay the disease onset and extend the survival of SOD1-G93A mice by ~10% (Apolloni et al., 2016). Spinal microglia taken from these mice during the symptomatic phase highlighted that clemastine also stimulated autophagic flux and decreased SOD-1 levels. Whether or not this effect was P2X7-dependent was not investigated in this study, but clemastine treatment was observed to enhance the expression of both P2X7 and P2Y12 (Apolloni et al., 2016). A study by Su et al., investigated the effect of clemastine on chronic unpredictable mild stress and depressive-like behavior in BALB/c mice (Su et al., 2018). Clemastine could limit IL-1β and TNF-α production in the hippocampus, suppress microglial M1-like activation, and improve astrocytic loss within the hippocampus (Su et al., 2018). They also show that clemastine treatment resulted in downregulation of hippocampal P2X7 expression (Su et al., 2018). However, whether these effects of clemastine were P2X7-dependent was not investigated.

Isatin (1*H*-ondole-2,3-dione) is found within plant and animal tissues, including human tissues (concentrations range from <0.1 to 10 µM). Several isatin derivatives exist with a diverse array of properties (anti-microbial, anti-convulsant, anti-inflammatory, and anti-cancer) and biological targets (proteases, kinases, and caspases). *N*-alkylated isatin derivatives which typically bind to tubulin to destabilise microtubules, were identified to enhance IL-1β secretion in a P2X7-dependent manner (Sluyter and Vine, 2016). In contrast to isatin or the parent synthetic molecule, 5, 7-dibromoisatin, the derivatives 5, 7-dibromo-*N*-(*p*-methoxybenzyl) isatin (NAI), and 3-4-[5,7-dibromo-1-(4-methoxybenzyl)-2-oxoindolin-3-ylidenamino]phenylpropanoic acid (NAI-imine) could enhance P2X7-induced IL-1β release from J774 mouse macrophages (Sluyter and Vine, 2016). However, neither NAI or NAI-imine potentiated ATP-induced responses including dye uptake and cell death suggesting that these chemicals may act downstream of the P2X7 receptor (Sluyter and Vine, 2016). Without further experimental evidence for their mechanism of activation, we have not classified the isatin derivatives as PAMs of P2X7.

Ivermectin, as previously discussed, is a commonly utilized PAM for P2X4. Challenging the selectivity of ivermectin for P2X4 within the family, Norenberg et al., demonstrated that ivermectin potentiated human P2X7 receptors but not murine P2X7 (Nörenberg et al., 2012). Utilizing electrophysiological and fluorometric methods, they observed potentiation of ATP-induced currents and Ca^2+^ influx in cells expressing human P2X7, but not rat or mouse P2X7 (Nörenberg et al., 2012). Notably, ivermectin could not potentiate other P2X7-driven functions such as YO-PRO-1 dye uptake (Nörenberg et al., 2012). Concentration-response experiments reveal that ivermectin has a minor effect on the EC_50_ value for ATP and can increase the maximum response (Nörenberg et al., 2012) suggesting classification as a mixed Type I/II PAM effect (**Table 1**). Ivermectin has been suggested to drive P2X4/P2X7/Pannexin-1 signaling to enhance numerous cell death pathways including apoptosis, necrosis, pyroptosis, and autophagy in cancer cells (Draganov et al., 2015).

Ginsenosides are steroid-like glycosides that are predominantly obtained from the roots of the plant genus *Panax ginseng*. Our lab first described four protopanaxadiol ginsenosides [Rb1, Rh2, Rd, and the metabolite compound K (CK)] that could potentiate ATP-activated P2X7 currents, dye uptake, and intracellular Ca^2+^ concentrations, with the most potent ginsenoside CK enhancing cell death toward a non-lethal concentration of ATP (Helliwell et al., 2015). Using molecular modeling and computational docking, we identified a novel binding site in the central vestibule region of human P2X7 (Bidula et al., 2019b) shared by other P2X receptors such as P2X4 (Dhuna et al., 2019). This predicted allosteric site involves amino acid residues Ser60, Asp318 and Leu320 in the β-strands connecting the orthosteric binding site to the transmembrane domains (Bidula et al., 2019b). This region is intimately involved in gating and more work now needs to be done to explore the mechanism of potentiation. Recently, we explored the effect of ginsenosides on P2X7-dependent cell death. High ATP (3 mM) was shown to induce an unregulated form of cell death in J774 mouse macrophages, while conversely, potentiation of a non-lethal concentration of ATP by ginsenoside CK could enhance apoptotic cell death in a caspase-dependent manner (Bidula et al., 2019a). In contrast to high ATP, the effect of ginsenoside CK could be reversed *via* the chelation of extracellular Ca^2+^, scavenging mitochondrial ROS, Bax inhibition, or by caspase inhibitors suggesting that different intracellular signaling events were involved following positive modulation (Bidula et al., 2019a).

Tenidap, a COX/5-LOX inhibitor and anti-inflammatory drug was discovered to be a potentiator of mouse P2X7 enhancing ATP-mediated cytotoxicity and Lucifer yellow dye uptake (Sanz et al., 1998). From the dose-response experiments performed in an LDH release assay (Sanz et al., 1998), it appears tenidap has mixed Type I/II PAM effects at P2X7 (**Table 1**). It is not known whether the effect of tenidap is restricted to mouse P2X7; no studies on human P2X7 can be found.

Polymyxin B, an antibiotic with bactericidal action against almost all Gram-negative bacteria, was identified to have potentiating action at P2X7 enhancing Ca^2+^ influx, membrane permeabilization, and cytotoxicity to low agonist concentrations (Ferrari et al., 2004). Interestingly, treatment with the irreversible inhibitor oxidised ATP or genetic ablation of P2X7 rendered cells insensitive to the synergistic effects of ATP and polymyxin B, but this effect was not replicated by the reversible P2X7 inhibitor KN-62 (Ferrari et al., 2004). Polymyxin B appears to left-shift the ATP concentration-response curve and increase the maximum response (Ferrari et al., 2004) thus it has mixed Type I/II PAM effects (**Table 1**). Polymyxin B nonapeptide, a derivative of polymyxin B lacking the N-terminal fatty amino acid 6-methylheptanoic/octanoic-Dab residue, did not have the same activity at P2X7 (Ferrari et al., 2007).

Agelasine and garcinoloic acid are two natural products capable of potentiating P2X7. Agelasines are bioactive 7,9-dialkylpurinium salts isolated from a marine sponge, whereas garcinolic acid is a xanthone derived from flowering plants of the species *Garsinia* (Fischer et al., 2014). Both agelasine and garcinolic acid compounds could potentiate P2X7 responses in HEK-293, A375 melanoma, and mouse microglial cells, but only garcinolic acid could significantly enhance P2X7-induced dye uptake (Fischer et al., 2014). Information regarding the type of PAM effect could not be extracted from the study as the effect of agelasine/garcinolic acid on the ATP concentration-response curves were not reported.

Other positive modulators of P2X7 include GW791343 (2- [(3,4- Difluorophenyl) amino]-*N*-[2-methyl-5-(1-piperazinylmethyl) phenyl]-acetamide trihydrochloride) a negative modulator at human P2X7, but a positive modulator at rat P2X7 (Michel et al., 2008a). Using ethidium uptake experiments to measure P2X7 responses, GW791343 increases potency and efficacy of the agonist BzATP at rat P2X7 expressed in HEK-293 cells (Michel et al., 2008a) suggesting a mixed Type I/II PAM effect (**Table 1**). Key structural differences exist between different species of P2X7 receptor and amino acid residue at position 95 is thought to be involved in coordinating GW791343 (Michel et al., 2008b). Anaesthetics such as ketamine, propofol, thiopental and sevofluranehave been identified as positive modulators of P2X7 in two independent studies (Nakanishi et al., 2007; Jin et al., 2013). Various phospholipids such as lysophosphatidylcholine, sphingophosphorylcholine, and hexadecylphosphorylcholine, can modulate the potency of ATP towards P2X7 (Michel and Fonfria, 2007). When used at sub-cytotoxic concentrations, each of these lipids could potentiate ethidium accumulation and P2X7-dependent IL-1β production from cells expressing recombinant or endogenous P2X7 respectively. However, when used at higher concentrations, the lipids induce an increase in intracellular Ca^2+^, radioligand binding, and cytotoxicity (Michel and Fonfria, 2007). Therefore, it is unclear whether the lipids are having a direct effect at P2X7 or simply inducing changes in the properties of the membrane itself. Phosphoinositides (anionic signaling phospholipids) can also positively modulate P2X7 *via* short, semi-conserved polybasic domain located in the proximal C-terminus of P2X subunits (Bernier et al., 2013). A single study has identified that P2X7 can be allosterically modulated by the glycosaminoglycan chains of CD44 proteoglycans present on Chinese hamster ovary (CHO) cells (Moura et al., 2015). The presence of these GAGs on the cell surface significantly increased the sensitivity of cells to ATP, potentiating Ca^2+^ influx and pore formation (Moura et al., 2015). Moreover, cells defective in GAG biosynthesis were protected from P2X7-dependent cell death (Moura et al., 2015). These works open up the possibility that allosteric modulation of P2X7 could occur *in vivo via* a multitude of mechanisms.

As the known number of positive allosteric modulators for P2X7 begins to increase, the question arises as to where positive modulation of P2X7 would be therapeutically beneficial. It has been well documented that P2X7 plays pivotal roles in immunity to infection and loss-of-function SNPs in P2X7 have proven deleterious. P2X7 has been demonstrated to provide immune protection towards viral (dengue), bacterial (chlamydia, periodontitis, tuberculosis), fungal (paracoccidioidomycosis), parasitic (leishmaniasis, trypanosomiasis, toxoplasmosis, amoebiasis, malaria), and helminth (schistosomiasis) infections. The causative agents of these diseases directly impact billions of people of worldwide and indirectly put many others at risk. Thus, the identification of novel positive allosteric modulators of P2X7 and further exploration into their biological effects would be significantly beneficial in the development of novel treatments to boost immune defences.

With the identification that positive modulation by ginsenoside CK could calibrate cell death responses of macrophages, promoting apoptotic cell pathways over lytic cell death pathways (Bidula et al., 2019a), we hypothesize that the ability to be able to pharmacologically promote these types of cell death pathways could be beneficial in the removal of pathogens, particularly intracellular pathogens such as mycobacteria and parasites which are not always effectively recognised by the immune system. Due to the role of P2X7 in regulating several cell death pathways, further investigation into whether other positive modulators could selectively promote alternative cell death pathways involved in the removal of pathogens could be important in the resolution of the infections listed above.

Another area in which positive modulation could be beneficial is in the treatment of cancers. P2X7 appears to participate in ameliorating myeloma, glioblastoma, non-small cell lung carcinoma, and melanoma, but the studies concerning cancer are often contradictory. However, a common characteristic among cancer cells is that many of them exhibit higher expression of P2X7 and that ATP at the tumor site is often abundant (Pellegatti et al., 2008; Roger et al., 2015). Stimulation of numerous cancer cell lines with high concentrations of ATP *in vitro* results in decreased viability of these cells. In cancer patients, it may be plausible to try and target cancer cells in two ways: administering a positive modulator to amplify the effects of enhanced local ATP concentrations around the tumor, activating P2X7 and inducing cell death or, alternatively, the use of an antibody-drug conjugate to target P2X7 specifically on these cells could be employed. Attaching a positive modulator to an antibody specific to P2X7, especially when targeting tumors with enhanced expression of P2X7, could deliver the modulator to where it is needed, amplify P2X7 responses on these cells, and induce death of the cancer cells. An issue arising from this method however, is if the cancer patient has any underlying pathologies associated with enhanced expression of P2X7, then targeting this receptor through antibody-drug conjugates might result in off-target effects and death of healthy cells.

## Conclusions

**Figure 2:**
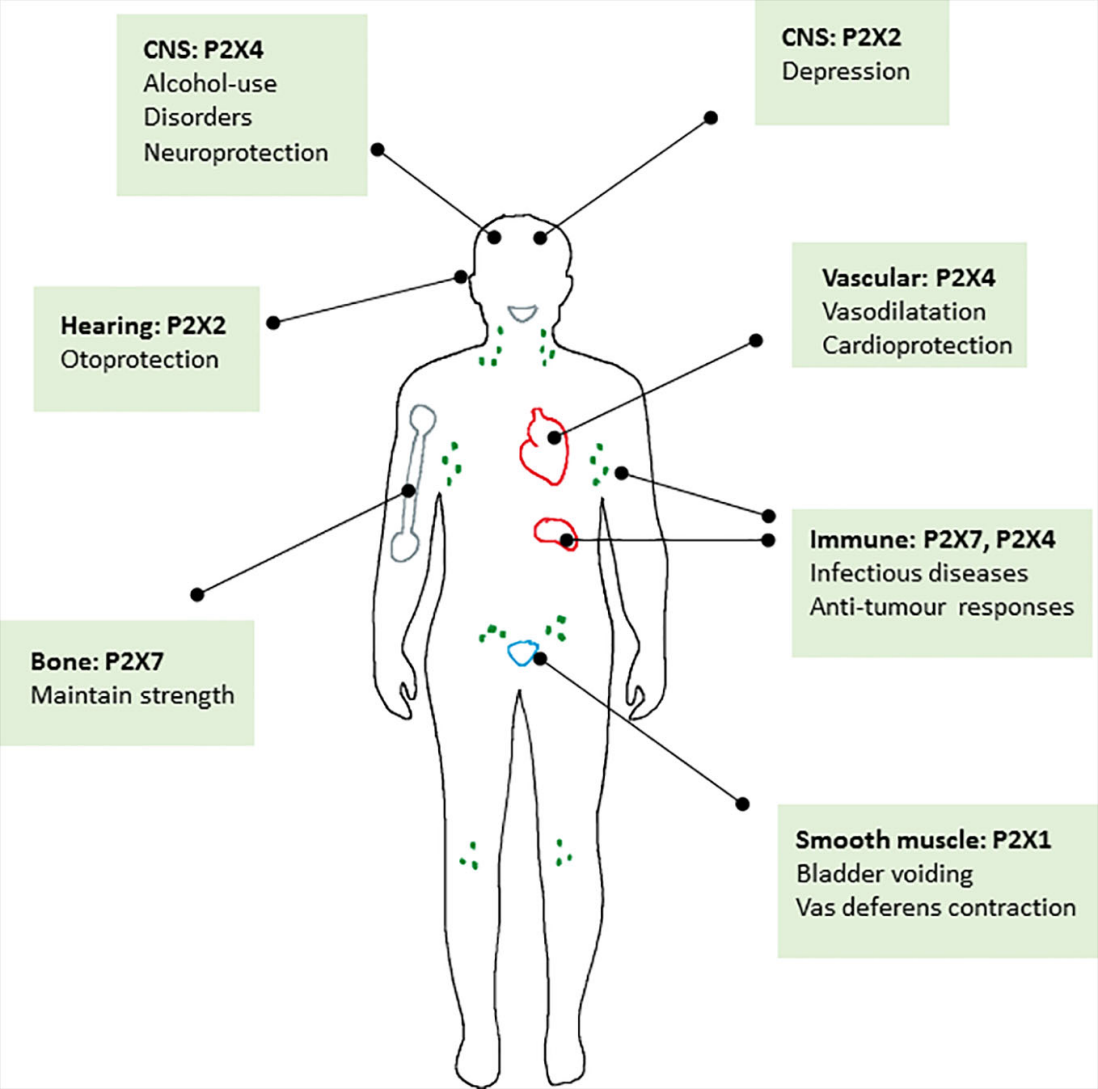
Schematic diagram summarising the major roles of P2X receptors in the body where positive allosteric modulators may have a therapeutic benefit.

Collectively it appears that there is good evidence that positive allosteric modulation of P2X2, P2X4, and P2X7 receptors may be of therapeutic benefit in a number of different conditions summarised in **Figure 2**. It is also clear that there may be endogenous molecules particularly in the central nervous system, that could act as positive modulators to enhance the action of the physiological agonist ATP (e.g., neurosteroids on P2X2 and P2X4). With regard to the question posed in the title, to inhibit or enhance, we have tried to present a balanced view of the knowledge surrounding the major physiological and pathophysiological roles for P2X receptors. There is a strong case for inhibition of several P2X receptors in a variety of diseases and clinical development of candidate compounds is in progress. However, this does not exclude the development of positive modulators for use in other disorders. We hope that we have highlighted these opportunities. Similar to other ligand-gated ion channels (NMDA receptors, nAchR) one challenge lies in drug selectivity for different forms of ion channels, typically subunit composition. With this in mind, knowing more about pharmacology of splice variants and polymorphic variants may be important for homomeric P2X receptors and understanding the differential pharmacology of heteromeric P2X receptors. With the advances in structural information and continued progress in allosteric binding pocket identification, plus access to the relevant animal models of disease, positive modulation of P2X receptors may become a fruitful area of research.

## Author Contributions

All authors contributed to the writing of the review. LS compiled and edited the final version.

## Funding

This work was funded by a BBSRC project grant (BB/N018427/1) and BBSRC DTP training grants 1794654 and 2059870.

## Conflict of Interest

The authors declare that the research was conducted in the absence of any commercial or financial relationships that could be construed as a potential conflict of interest.
